# [Ag(I)(Et_2_PCH_2_CH_2_PPh_2_)_2_]NO_3_: An Antimitochondrial Silver Complex

**DOI:** 10.1155/MBD.1995.111

**Published:** 1995

**Authors:** Susan J. Berners-Price, Doreen C. Collier, Muhammed A. Mazid, Peter J. Sadler, Rodney E. Sue, David Wilkie

**Affiliations:** 1 School of Science Griffith University Queensland Nathan 4111 Australia; 2 Department of Biology University College Gower St. London WCIE 6BT United Kingdom; 3 School of Chemistry and Applied Chemistry University of Wales College of Cardiff Cardiff CF1 3TB United Kingdom; 4 Department of Chemistry Birkbeck College 29 Gordon Sq. London WC1H 0PP United Kingdom

## Abstract

The silver(I) complex [Ag(eppe)_2_]NO_3_ (eppe = Et_2_PCH_2_CH_2_PPh_2_) is shown by X-ray crystallography to be tetrahedral with Ag - PEt_2_  and Ag - P Ph_2_  bond lengths of 2.482 and 2.518 Å, respectively. The complex is selectively antimitochondrial and inhibits the growth of a number of yeast strains in non-fermentable media at concentrations as low as 2.5 μΜ and induces the mitochondrial mutation *petite* The effect is largely reversed by the presence of aspirin. The complex is shown to be stable in the cell culture media and in the presence of glutathione, but readily reacts with disulfides of oxidized glutathione and serum albumin. Surprisingly, neither [Au(eppe)_2_]Cl nor [Au(eppe)_2_]Cl  (dppe = Ph_2_PCH_2_CH_2_PPh_2_) showed any mitochondrial selectivity in the same screening protocol.


### $[Ag(I)(Et_2PCH_2CH_2PPh_2)_2]NO_3$: AN ANTIMITOCHONDRIAL SILVER COMPLEX

Susan J. Berners-Price,[1] Doreen C. Collier,[2] Muhammed A. Mazid,[3]
Peter J. Sadler,[4] Rodney E. Sue[4] and David Wilkie[2]

[1] School of Science, Griffith University, Nathan, Queensland, 4111, Australia
[2] Department of Biology, University College, London, Gower St., London WC1E 6BT, U.K.
[3] School of Chemistry and Applied Chemistry, University of Wales College of Cardiff, Cardiff CF1 3TB, U.K.
[4] Department of Chemistry, Birkbeck College, 29 Gordon Sq., London WC1H 0PP, U.K.

### ABSTRACT

The silver(I) complex $[Ag(eppe)_2]NO_3$ (eppe = $Et_2PCH_2CH_2PPh_2$) is shown by X-ray crystallography to be tetrahedral with Ag - $PEt_2$ and Ag - P $Ph_2$ bond lengths of 2.482 and 2.518 Å, respectively. The complex is selectively antimitochondrial and inhibits the growth of a number of yeast strains in non-fermentable media at concentrations as low as 2.5 μM and induces the mitochondrial mutation *petite*. The effect is largely reversed by the presence of aspirin. The complex is shown to be stable in the cell culture media and in the presence of glutathione, but readily reacts with disulfides of oxidized glutathione and serum albumin. Surprisingly, neither $[Au(eppe)_2]Cl$ nor $[Au(dppe)_2]Cl$ (dppe = $Ph_2PCH_2CH_2PPh_2$) showed any mitochondrial selectivity in the same screening protocol.

### INTRODUCTION

The potent antibacterial properties of silver compounds have been known for some time. For example, silver sulfadiazene has been used as a topical burn treatment to prevent bacterial infection.[1,2] and has been reported to have antifungal, antiherpes virus and antitreponemal activity.[3] $[Ag(dppe)_2]NO_3$ and $[Ag(depe)_2]NO_3$ were also found to have antibacterial and antifungal activity against *C. albicans* with activity comparable to fungizone, in a defined medium.[4] However, the antifungal activity was lost in Sabouraud's broth medium, as was the antitumour activity in media containing fetal calf serum. This illustrates a general problem with the development of silver compounds as drugs, *viz.* their reactivity in physiological fluids, with strong binding to components of the fluid, or sometimes precipitation of Ag(I) salts such as AgCl preventing uptake of silver. The success of silver sulfadiazene, which is polymeric in the solid state with distorted trigonal bipyramidal coordination geometry,[2] is attributed to the slow release of Ag(I) ions which function as the bactericide at the wound site.[5] The development of resistance to antibacterial silver preparations appears to be related to the ability of resistant bacteria to bind silver more strongly than chloride,[6] and hence the silver is unavailable to these bacteria.

Of the 100 silver compounds reported to have been tested by the National Cancer Institute for anticancer activity, only 5 had marginal activity at best.[7] Interestingly, the binuclear silver complex $[(\mu\text{-dppe})(AgNO_3)_2]$ was inactive against P388 leukemia cells,[8] which may be due to the increased kinetic reactivity of the linear silver complex compared to the tetrahedral complex. In an attempt to overcome these difficulties, some success has been achieved in reducing the kinetic reactivity of silver(I) complexes by imposing tetrahedral geometry at the metal centre, which tends to shield the metal from competing ligands,[7] thereby potentially allowing better access of the silver complex to the target site. Tetrahedral Ag(I) diphosphine complexes have been shown to be both potent cytotoxic and antitumour agents.[7] The silver(I) complexes $[AgL_2]NO_3$ {L = $Ph_2PCH_2CH_2PPh_2$ (dppe); $Et_2PCH_2CH_2PEt_2$ (depe); *cis*-$Ph_2PCH{=}CHPPh_2$ (dppey)} are cytotoxic towards B16 melanoma cells *in vitro* with $IC_{50}$ values of *ca.* 4 μM ($IC_{50}$ is the concentration required to inhibit cloning efficiency by 50 %), which is comparable to the extensively studied Au(I) analogue. The complexes were also active against intraperitoneal P388 leukemia in mice and M5076 reticulum sarcoma.[4] Cancer cells frequently possess respiratory defective mitochondria,[9] and it has been suggested that these may prove to be potential targets for novel antitumour agents.[10] A screening protocol for potential anticancer drugs based on the yeast mitochondrial system has been reported.[11] The yeast cell is now widely accepted as a suitable model of the eukaryotic cell with mitotic and meiotic cycles and with mitochondria similar in all fundamental respects to those of mammalian cells.

We now report that the tetrahedral silver diphosphine complex $[Ag(eppe)_2]NO_3$ (eppe = $Et_2PCH_2CH_2PPh_2$) shows selective, primary antimitochondrial activity in yeast cells, and also discuss the chemistry of this complex in solution and in the solid state.

### MATERIALS AND METHODS

### *Chemicals*

$Et_2PCH_2CH_2PPh_2$ was purchased from Strem Chemical Inc.. All other chemicals were supplied by the Aldrich Chemical Co. and were used without further purification. Bovine serum albumin (fraction V, fatty acid free) was obtained from Boehringer Mannheim GmbH.

### *Synthesis*

*Bis(1-(diethylphosphino)-2-(diphenylphosphino)ethane)silver(I) nitrate.*[12]

Under argon, silver nitrate (73 mg, 0.4 mmol) was dissolved in a solution of 1-(diethylphosphino)-2-(diphenylphosphino)ethane (260 mg, 0.9 mmol) in chloroform (10 mL; degassed by purging with argon). After 2 h all the material had dissolved, and the mixture was stirred overnight. The mixture was filtered and the solvent removed *in vacuo* to give a colourless oil which was crystallized by trituration with ice-cold ether (286 mg, 86 %, m.p. 134-152 °C). Calculated for $C_{36}H_{48}AgNO_3P_4$ - C: 55.8 %, H: 6.3 %, N: 1.8 %, P: 16.0 %; Found - C: 55.4 %, H: 6.4 %, N: 1.6 %, P: 15.6 %

### *NMR Measurements*

$^{31}P\{^1H\}$-nmr spectra were obtained on a JEOL GSX-500 spectrometer operating at 202.35 MHz, or a JEOL GSX-270 spectrometer operating at 109.35 MHz, with broad band proton decoupling. Unless otherwise noted, samples were contained in 5 mm tubes and spectra were recorded at ambient temperature (ca. 22 °C) except for the reactions involving serum albumin, which were recorded at 37 °C. Typical accumulation conditions were 3 - 18 kHz spectral width, 0.7 - 1.4 s acquisition time, 15 - 30 μs pulse width, 2 s relaxation delay and 16 K data points. All spectra were referenced to 85 % $H_3PO_4$ (external). $^1H$-nmr spectra were recorded on a JEOL GSX-270 spectrometer operating at 270.16 MHz, typical accumulation conditions were a 3.2 kHz spectral width, 2.5 s acquisition time, 5 μs pulse width, 2 s recycle delay and 16 K data points. $^1H$ spectra were referenced to TSP *via* residual solvent resonances.

Variable temperature $^{31}P$ nmr spectra were collected for 65 mM solutions of $[Ag(eppe)_2]NO_3$ in $CDCl_3$. For reactivity studies, typically the complex (*ca.* 5 mg) was dissolved in 200 μL dmso, and was added to 500 μL $D_2O$, 1 M NaCl, glutathione (oxidized or reduced) solution in $D_2O$, albumin solution in deuterated phosphate solution or yeast culture media, to give a final concentration of the complex of *ca.* 9 mM. Solutions of glutathione (oxidized or reduced) were prepared by dissolving the required amount (1, 4 or 8 equivalents) in $D_2O$ and were studied directly ($pH^*$ *ca.* 5) or with adjustment of $pH^*$ to *ca.* 7 with 0.2 M NaOD. Bovine serum albumin solutions were prepared by dissolving the protein in 10 mM phosphate solution in $D_2O$ (final $pH^*$ *ca.* 6.9) to give protein concentrations of 0.63 or 2.0 mM. Yeast extracts were prepared as described for the biological studies and spectra of these solutions were collected without deuterium lock. Solutions of yeast extracts were pale yellow, and all other solutions were colourless, After addition of the silver complex, samples were clear, or slightly cloudy, and were suitable for examination by $^{31}P\{^1H\}$ nmr, except albumin samples, which immediately yielded a thick white precipitate. These samples were centrifuged and the supernatants studied by $^{31}P\{^1H\}$ nmr.

*Crystallography*

Colourless prisms suitable for single crystal X-ray diffractometry were obtained upon standing a 9 mM solution of the $[Ag(eppe)_2]NO_3$ in dmso: $D_2O$ (2:5 v/v).for one week.

*Crystal Data*: $C_{36}H_{48}AgNO_3P_4$, $M$ = 774.5, triclinic, $a$ = 13.06(2), $b$ = 16.60(2), $c$ = 19.18(2) Å $\alpha$ = 105.01(3), $\beta$ = 91.77(5), $\gamma$ = 90.27(4)°, space group $P\bar{1}$, Z = 4, U = 3770(9) Å$^3$, $D_c$ = 1.365 Mg m$^{-3}$, $F(000)$ = 1608, $\mu$(Mo-$K_\alpha$) = 20.285 cm$^{-1}$.

*Data Collection.* Unit cell dimensions and intensity data were obtained at 293 K using an Enraf-Nonius diffractometer and area detector with graphite monochromated Mo-$K_\alpha$ radiation, following previously described procedures[13] (D = 50 mm, $2\theta_D$ = 23°). A total of 12000 reflections were measured, of which 8740 were unique.

*Biology*

The yeast *Saccharomyces cerevisiae* is a faculative anaerobe and as such can grow in the absence of mitochondrial respiratory function provided the glycolytic cycle (glucose metabolism) is operative in generating ATP. Haploid yeast strains of *Saccharomyces cerevisiae* were used from a standard collection in our laboratory. The yeast culture media contained 1% yeast extract (Difco) and either 2% glucose (YED) or 4% glycerol (YEG) as the carbon and energy sources. Where solid medium was required, 1 % agar (Difco) was added to the medium.

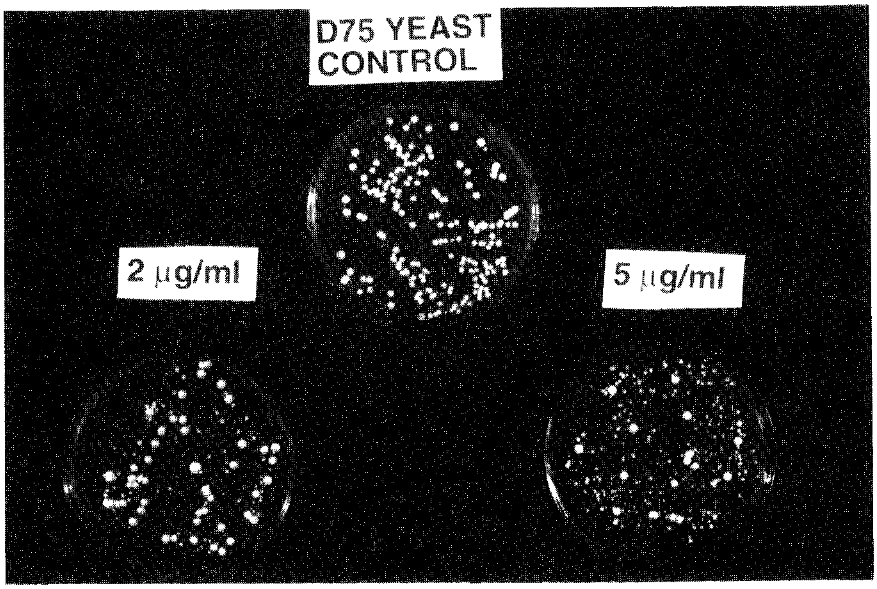

**Figure 1.** Cultures of yeast strain D75 in fermentable (YED) medium showing dependence on dose of $[Ag(eppe)_2]NO_3$. The small white colonies are those of the *petite* mutation. The larger colonies are normal cells.

Drop inocula of cells from 15 strains of yeast were plated on either YEG- or YED-agar media containing [Ag(eppe)$_2$]NO$_3$. Typically the complex was dissolved in dmso and was added to the medium to the required concentrations (2.6, 6.5 and 13 μM). Control tests showed dmso had little or no effect on the growth of cells at the concentrations used. Growth was recorded after 2 days incubation at 30 °C. Treated cultures growing on glucose medium were sampled and cells plated on YED-agar. Colonies of the mitochondrial mutation *petite*[14] that appeared were identified as small white colonies compared to the larger, creamy colonies of unaffected strains, making scoring unambiguous (Figure 1). Respiratory deficiency was verified by failure to grow when transferred to YEG medium. The effect of aspirin was studied in both the YEG and YED media at a concentration of 13 μM [Ag(eppe)$_2$]NO$_3$ (10 μg/mL) and 5.4 mM aspirin (1 mg/mL) in the media, and were scored after 5 days. Toxicity was measured in terms of cell death.

### RESULTS

### *Chemical Studies*

The light stable silver complex, [Ag(eppe)$_2$]NO$_3$, was prepared by a similar procedure to that reported earlier,[12] and was found to be stable in dmso:water (2:5 v/v) solution for at least 24 hours by $^{31}$P{$^1$H} nmr. The spectrum at 295 K consists of a complicated multiplet pattern centered at 2.3 ppm in this solvent, and 2.0 ppm in CDCl$_3$. The mulitplet structure was resolved by cooling the solution in CDCl$_3$ to 258 K. The spectrum of the complex in 1 M NaCl remained unchanged for at least several hours.

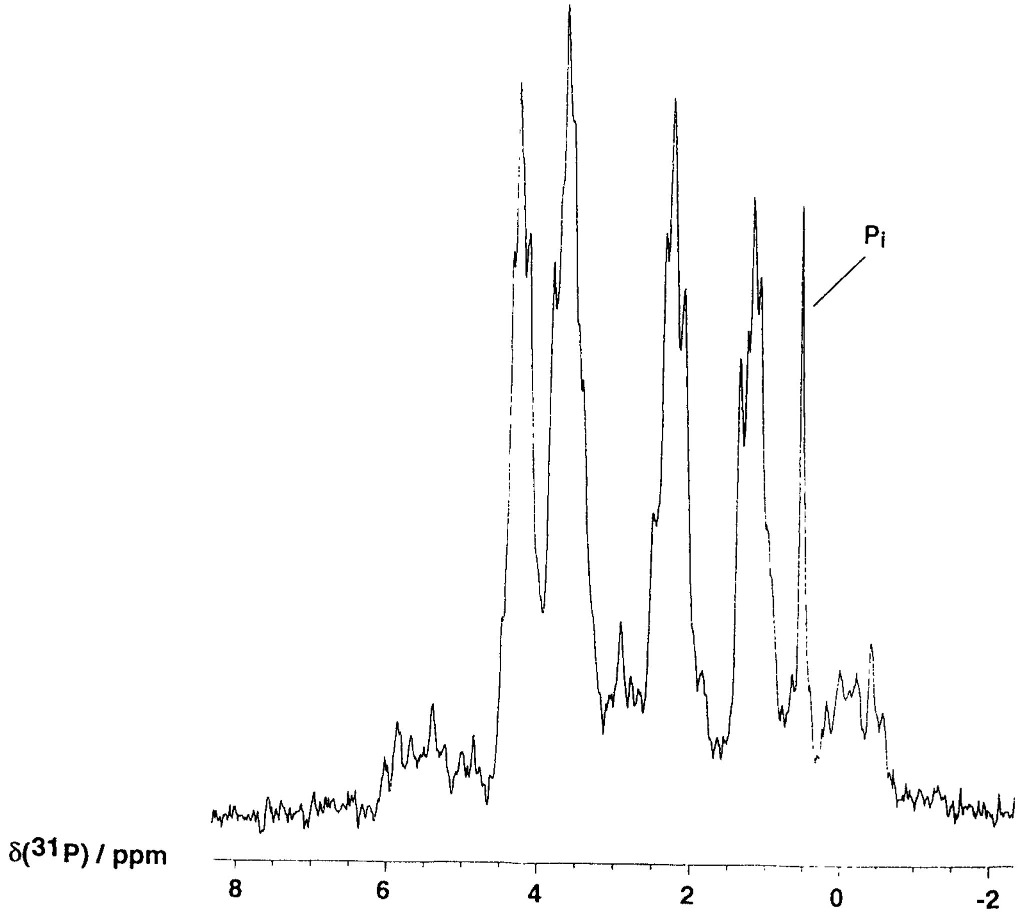

**Figure 2.** $^{31}$P{$^1$H} nmr spectrum of [Ag(eppe)$_2$]NO$_3$ in YEG medium, the spectrum is identical to that of the complex in the absence of culture medium.

No changes were evident in the spectra of the complex in both the fermentable and non-fermentable yeast extracts, although an additional resonance at *ca.* 0.5 ppm was observed, assignable to phosphate in the media (Figure 2). Spectra from mixtures of the complex and reduced glutathione at pH* 5 or 7 showed the presence of unreacted silver complex, in addition to two minor resonances at 62.5 and 40.3 ppm, attributable to traces of $Et_2P(O)CH_2CH_2P(O)Ph_2$. When a solution of $[Ag(eppe)_2]NO_3$ in dmso was added to 0.63 mM or 2.0 mM solutions of bovine serum albumin in 10 mM phosphate solution (pH* 7), a thick white precipitate formed immediately, which was removed by centrifugation. The spectrum of the supernatant showed two doublets at 62.5 and 40.3 ppm J = 53 Hz, consistent with the formation of $Et_2P(O)CH_2CH_2P(O)Ph_2$. On addition of the complex to a solution containing 1 or 4 mol equivalents of oxidized glutathione, $^{31}P\{^1H\}$ nmr spectroscopy indicated the presence of both the diphosphine dioxide, as for albumin, and a multiplet centred at 2.0 ppm, assigned to unreacted silver complex. Complete reaction of the silver complex was observed when 8 mol equivalents of oxidized glutathione were used. $^1H$ nmr spectra indicated the presence of reduced glutathione in the reaction mixture.

*Molecular Structure*

The single crystal X-ray structure was solved by the heavy atom method,[16] and was refined by full matrix least squares methods (SHELX-93).[17] Absorption corrections were applied at the isotropic refinement stage using the DIFABS procedure,[18] adapted for FAST geometry.[19] H atoms were allowed to ride on their parent carbon atoms in their calculated positions ($C_{alkyl}$ - H = 0.97, $C_{aryl}$ - H = 0.93 Å). The final R1 and WR2 values were 0.0472 and 0.1229, respectively. Non-hydrogen atom coordinates and equivalent isotropic thermal parameters are displayed in Table 1.

**Table 1.** Atomic coordinates (x $10^4$) and equivalent isotropic displacement parameters ($Å^2$ x $10^3$) for $C_{36}H_{48}AgNO_3P_4$. U(eq) is defined as one third of the trace of the orthogonalized $U_{ij}$ tensor.

| | x | y | z | U(eq) |
|---|---|---|---|---|
| Ag(l) | 2281(1) | 2091(1) | 4604(1) | 21(1) |
| P(11) | 1169(1) | 2543(1) | 5686(1) | 20(1) |
| P(12) | 2955(1) | 3124(1) | 3919(1) | 23(1) |
| P(13) | 838(1) | 1085(1) | 4053(1) | 22(1) |
| P(14) | 4095(1) | 1737(1) | 4802(1) | 29(1) |
| N(11) | 2706(3) | 8645(3) | 4887(2) | 29(1) |
| O(11) | 2634(3) | 8397(3) | 5456(2) | 43(1) |
| O(12) | 2813(3) | 9453(3) | 4917(2) | 44(1) |
| O(13) | 2672(3) | 8076(3) | 4289(2) | 50(1) |
| C(11) | 1923(3) | 1130(4) | 6172(3) | 25(1) |
| C(12) | 2033(4) | 589(4) | 6646(3) | 27(1) |
| C(13) | 1594(4) | 834(4) | 7318(3) | 29(1) |
| C(14) | 1032(4) | 1603(4) | 7508(3) | 28(1) |
| C(15) | 903(3) | 2137(4) | 7035(2) | 25(1) |
| C(16) | 1361(3) | 1901(3) | 6361(2) | 21(1) |
| C(17) | 1694(4) | 4075(4) | 6788(3) | 33(1) |
| C(18) | 1660(5) | 4962(4) | 7146(3) | 47(2) |
| C(19) | 929(5) | 5499(5) | 6934(4) | 53(2) |
| C(110) | 236(5) | 5144(5) | 6375(3) | 47(2) |
| C(111) | 267(4) | 4250(4) | 6024(3) | 35(1) |
| C(112) | 996(4) | 3686(4) | 6214(3) | 27(1) |
| C(113) | 2310(4) | 4684(4) | 4870(3) | 34(1) |
| C(114) | 2209(4) | 5591(4) | 5137(3) | 39(1) |
| C(115) | 2726(4) | 6168(4) | 4835(3) | 38(1) |
| C(116) | 3364(4) | 5836(4) | 4261(3) | 38(2) |
| C(117) | 3485(4) | 4926(4) | 3999(3) | 32(1) |
| C(118) | 2955(4) | 4322(4) | 4295(2) | 25(1) |
| C(119) | 2041(3) | 3654(4) | 2728(3) | 27(1) |

**Table 1 (cont.).** Atomic coordinates (x $10^4$) and equivalent isotropic displacement parameters ($Å^2$ x $10^3$) for $C_{36}H_{48}AgNO_3P_4$.

| | x | y | z | U(eq) |
|---|---|---|---|---|
| C(120) | 1672(4) | 3485(4) | 2011(3) | 32(1) |
| C(121) | 1780(4) | 2665(4) | 1537(3) | 29(1) |
| C(122) | 2250(4) | 1985(4) | 1773(3) | 33(1) |
| C(123) | 2600(4) | 2150(4) | 2486(3) | 32(1) |
| C(124) | 2516(3) | 2976(4) | 2970(2) | 24(1) |
| C(125) | -117(3) | 2143(4) | 5300(3) | 30(1) |
| C(126) | 75(4) | 1207(4) | 4796(3) | 28(1) |
| C(127) | 4807(3) | 2611(4) | 4512(3) | 28(1) |
| C(128) | 4326(3) | 2826(4) | 3825(3) | 29(1) |
| C(129) | 74(4) | 1342(4) | 3315(3) | 32(1) |
| C(130) | -280(5) | 2308(4) | 3483(3) | 44(2) |
| C(131) | 966(4) | -113(4) | 3761(3) | 34(1) |
| C(132) | 1646(6) | -443(6) | 3118(4) | 76(2) |
| C(133) | 4627(4) | 1881(5) | 5734(3) | 52(2) |
| C(134) | 4259(5) | 2679(5) | 6277(3) | 59(2) |
| C(135) | 4680(4) | 689(4) | 4342(3) | 44(2) |
| C(136) | 4625(5) | 478(5) | 3526(3) | 59(2) |
| Ag(2) | 2964(1) | 7526(1) | 112(1) | 21(1) |
| P(21) | 1881(1) | 6297(1) | 367(1) | 19(1) |
| P(22) | 4157(1) | 7224(1) | -929(1) | 19(1) |
| P(23) | 1310(1) | 8292(1) | 88(1) | 25(1) |
| P(24) | 4479(l) | 8299(1) | 843(1) | 21(1) |
| N(21) | 7675(4) | 9246(4) | 393(2) | 39(1) |
| O(21) | 7084(3) | 9740(3) | 161(3) | 58(1) |
| O(22) | 8221(4) | 8740(4) | -34(3) | 84(2) |
| O(23) | 7699(4) | 9222(5) | 1025(3) | 91(2) |
| C(21) | 2352(4) | 6984(4) | 1839(3) | 26(1) |
| C(22) | 2293(4) | 7062(4) | 2574(3) | 32(1) |
| C (23) | 1612(4) | 6525(4) | 2818(3) | 32(1) |
| C(24) | 989(4) | 5925(4) | 2318(3) | 29(1) |
| C(25) | 1041(3) | 5847(4) | 1584(3) | 25(1) |
| C(26) | 1742(3) | 6370(3) | 1332(2) | 20(1) |
| C(27) | 1668(3) | 4765(4) | -803(3) | 26(1) |
| C(28) | 1735(4) | 3882(4) | -1138(3) | 31(1) |
| C(29) | 2079(3) | 3271(4) | -763(3) | 30(1) |
| C(210) | 2366(4) | 3598(4) | -31(3) | 29(1) |
| C(211) | 2298(3) | 4487(4) | 312(3) | 26(1) |
| C(212) | 1928(3) | 5105(3) | -64(2) | 20(1) |
| C(213) | 5549(4) | 5964(4) | -1726(2) | 28(1) |
| C(214) | 5900(4) | 5118(4) | -1997(3) | 33(1) |
| C(215) | 5420(4) | 4402(4) | -1843(4) | 51(2) |
| C(216) | 4576(5) | 4559(5) | -1400(5) | 80(3) |
| C(217) | 4226(4) | 5405(4) | -1131(4) | 48(2) |
| C(218) | 4692(3) | 6129(4) | -1287(3) | 24(1) |
| C(219) | 3514(4) | 8490(4) | -1618(3) | 31(1) |
| C(220) | 3144(4) | 8803(5) | -2189(3) | 42(2) |
| C(221) | 3024(4) | 8233(5) | -2877(3) | 45(2) |
| C(222) | 3271(4) | 7355(5) | -2990(3) | 41(2) |
| C(223) | 3658(4) | 7032(4) | -2417(3) | 29(1) |
| C(224) | 3788(3) | 7599(3) | -1725(2) | 20(1) |
| C(225) | 578(3) | 6577(4) | 81(3) | 24(1) |
| C(226) | 367(3) | 7554(3) | 357(3) | 25(1) |
| C(227) | 5529(3) | 7847(4) | 242(2) | 24(1) |

**Table 1 (cont.).** Atomic coordinates (x $10^4$) and equivalent isotropic displacement parameters ($Å^2$ x $10^3$) for $C_{36}H_{48}AgNO_3P_4$.

| | x | y | z | U(eq) |
|---|---|---|---|---|
| C(228) | 5300(3) | 7917(4) | -529(2) | 26(1) |
| C(229) | 820(5) | 8426(6) | -780(4) | 76(3) |
| C(230) | 945(6) | 7613(7) | -1412(4) | 90(4) |
| C(231) | 1074(7) | 9348(6) | 705(6) | 117(4) |
| C(232) | 680(14) | 9603(8) | 1262(6) | 249(11) |
| C(233) | 4903(4) | 8114(4) | 1722(3) | 30(1) |
| C(234) | 5955(4) | 8482(5) | 2034(3) | 40(2) |
| C(235) | 4661(4) | 9501(4) | 966(3) | 28(1) |
| C(236) | 4027(4) | 10071(4) | 1554(3) | 44(2) |

**Table 2.** Selected bond lengths (Å) and angles for each molecule in the asymmetric unit of $[Ag(eppe)_2]NO_3$.

| | molecule 1 | molecule 2 |
|---|---|---|
| *Bond lengths* | | |
| Ag - P(1) | 2.520(3) | 2.533(3) |
| Ag - P(2) | 2.501(2) | 2.519(3) |
| Ag - P(3) | 2.472(3) | 2.477(3) |
| Ag - P(4) | 2.475(4) | 2.502(3) |
| P(1) - C(25) | 1.853(5) | 1.860(5) |
| P(2) - C(28) | 1.855(5) | 1.862(5) |
| P(3) - C(26) | 1.858(5) | 1.856(5) |
| P(4) - C(27) | 1.857(5) | 1.840(5) |
| C(25) - C(26) | 1.531(8) | 1.508(8) |
| C(27) - C(28) | 1.557(7) | 1.531(7) |
| *Bond Angles* | | |
| P(2) - Ag - P(1) | 124.84(8) | 121.19(7) |
| P(3) - Ag - P(1) | 85.04(10) | 84.67(11) |
| P(4) - Ag - P(1) | 118.25(9) | 127.07(7) |
| P(3) - Ag - P(2) | 118.19(8) | 122.66(8) |
| P(4) - Ag - P(2) | 86.14(8) | 84.90(11) |
| P(4) - Ag - P(3) | 129.36(9) | 121.28(10) |
| C(25) - P(1) - Ag | 102.4(2) | 101.8(2) |
| C(26) - P(3) - Ag | 104.0(2) | 104.0(2) |
| C(27) - P(4) - Ag | 103.1(2) | 100.9(2) |
| C(28) - P(2) - Ag | 103.0(2) | 102.1(2) |
| C(26) - C(25) - P(1) | 111.4(4) | 111.7(3) |
| C(25) - C(26) - P(3) | 114.5(3) | 114.4(3) |
| C(28) - C(27) - P(4) | 113.4(3) | 111.4(3) |
| C(27) - C(28) - P(2) | 112.9(3) | 110.4(3) |

The complex crystallizes with 2 molecules in the asymmetric unit. The Ag in each case is coordinated to four phosphorus atoms with distorted tetrahedral geometry at silver. The Ag - $PEt_2$ bond lengths of 2.482 Å (av) are slightly shorter than the Ag - $PPh_2$ lengths of 2.518 Å (av). The intra-ligand $Et_2P$-Ag-$PPh_2$ bond angles are all approximately 85°. Selected bond lengths and angles for both molecules in the asymmetric unit are displayed in Table 2. Thermal parameters indicate minor disorder in one of the ethyl groups of one molecule. Figure 3 depicts a projection of the asymmetric unit down the x-axis.

*Biological Studies*

A dose-dependent response was observed in the yeast cultures. Growth was inhibited on the non-fermentable glycerol (YEG) medium at concentrations as low as 2.5 μM (2 μg/mL) but growth proceeded in glucose containing cultures in the presence of the complex. Cells plated on the fermentable D-glucose (YED) medium showed induction of the mitochondrial mutation *petite* in most strains (Figure 1). Although growth was inhibited on the YEG medium in the presence of the complex, normal growth was observed when aspirin was also present. On the YED medium, growth was observed in the presence or absence of aspirin.

### DISCUSSION

*Structure and Reactivity of [Ag(eppe)$_2$]$NO_3$*

In the solid state, [Ag(eppe)$_2$]$NO_3$ crystallizes with two molecules of opposite chirality in the asymmetric unit. In each case, the silver possesses flattened tetrahedral geometry. There are two types of Ag - P bond in the complex, with the Ag - $PPh_2$ bonds (2.501 - 2.533 Å) being slightly longer than the Ag - $PEt_2$ bonds (2.472 - 2.502 Å), $PEt_2$ being the more basic phosphorus in the ligand.[20] The structure of a number of tetrahedral silver complexes with phosphines have been reported, and the Ag - $PEt_2$ distances are similar to those observed for [Ag($Me_2PCH_2CH_2PMe_2$)$_2$]$BPh_4$ (2.496 Å, average).[21] By comparison, the longer Ag - $PPh_2$ distances in [Ag(eppe)$_2$]$NO_3$ are similar to the Ag - P distance reported forthe related dppe complexes such as [Ag(dppe)$_2$]$NO_3$ (2.513 Å, average)[22] and the dimeric complex [$Ag_2$(dppe)$_4$]($NO_3$.$CH_3OH$)$_2$, which contains both bridging and chelating ligands (2.544 Å, average).[23] Both types of bond length are shorter than the Ag - P distance of 2.64 - 2.75 Å reported for a number of $[Ag(PPh_3)_4]^+$ complexes, although it is thought that the Ag - P distance is lengthened in these cases due to steric crowding of the phenyl rings, which is less severe in the bidentate diphosphines.[24] Curiously the related complex, [Ag(*cis*-$Ph_2PCH{=}CHPPh_2$)$_2$][$SnPh_3(NO_3)_2$]has a shorter Ag -P distance of 2.473 Å (average),[25] despite the lower $pK_a$ for this diphosphine.[20] The restricted bite of the eppe diphosphine ligand imposes a small intra-ligand $Et_2P$ - Ag - $PPh_2$ angle (84.5 - 86.5°) on the complex and results in opening of the inter-ligand P - Ag - P angles. This is the major cause of distortions from ideal geometry, as for other silver complexes with bidentate phosphines.[21-23, 25]

In chloroform solution, the complicated splitting pattern of the $^{31}P\{^1H\}$ nmr spectrum is consistent with the coupling of 4 magnetically inequivalent phosphorus atoms coupled to the two spin -1/2 isotopes $^{107}Ag$ and $^{109}Ag$ (51 % and 49 %, respectively) in an $[AA'BB'X]_2$ spin system. In an earlier study[12] we estimated the two J($^{109}Ag$-$^{31}P$) coupling constants to be -290 and -232 Hz from analysis of a $^{109}Ag$ spectrum recorded by the use of a $^{109}\{^{31}P\}$ DEPT sequence. The presence of the two unequal Ag - P couplings is consistent with the unequal Ag - P bond lengths shown in the x-ray structure, and we now assign the larger (-290 Hz) coupling constant to the shorter Ag - $PEt_2$ bonds andf the -232 Hz coupling to the Ag - $PPh_2$ bonds. The complex undergoes dynamic ligand exchange and a metal centred inversion process, so that in $CDCl_3$ solutions above 235 K only one set of proton nmr resonances were observed for both the ethyl and phenyl groups in the two chiral molecule.[26]

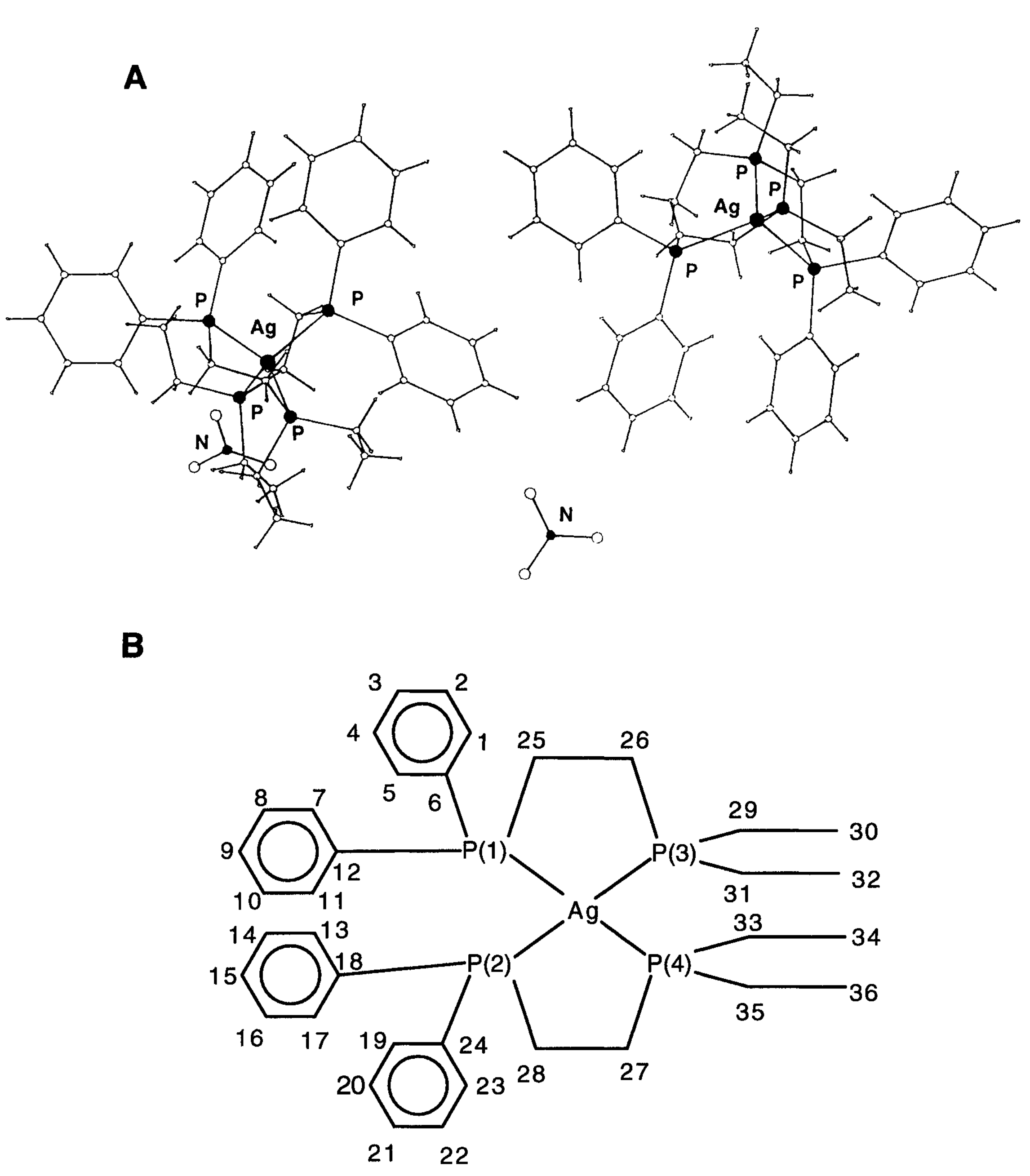

**Figure 3.** **A**: Molecular structure of both $[Ag(eppe)_2]NO_3$ molecules in the asymmetric unit. Each Ag has flattened tetrahedral geometry, the molecules differ in chirality and in orientation of the substituents at phosphorus.

**B**: Numbering system, numbers are prefaced by 1 for molecule 1, and 2 for molecule 2.

The studies reported here show that $[Ag(eppe)_2]NO_3$ is stable in aqueous solution as well as in 1 M NaCl solution, although in analogous Ag(I) complexes with monodentate phosphines, chloride ion binds to silver with the exclusion of a fourth ligand.[27] This is perhaps a reflection of the enhanced thermodynamic and kinetic stability of the *bis*-chelated silver diphosphine complexes compared to complexes with monodentate phosphines. The complex also remains intact in media containing either glycerol or glucose yeast extracts. Therefore it is likely that the tetrahedral complex is responsible for the biological activity. Further reactivity studies showed that the complex was not destroyed by reduced glutathione in aqueous media over several hours, indicating slow or little reaction with this thiol. However, the complex did react rapidly with bovine serum albumin, giving rise to the immediate formation of a thick white precipitate, presumably denatured protein. The denatured protein may contain reduced disulfide bridges since the oxidized ligand, $Et_2P(O)CH_2CH_2P(O)Ph_2$, was detected as product by comparison with known chemical shifts.[15] Serum albumin is known to oxidize phosphines. The auranofin analogues $[(Et_3P)_2Au]Cl$ and $[Pr^i{}_3PAuSATg]$, (HSATg = 2,3,4,6-tetra-O-acetyl-1-thio-β-D-glucose) both react with the disulfide linkages of albumin to give phosphine oxide and free thiol groups.[28] $[Au(eppe)_2]Cl$ is also oxidized by serum albumin, giving $Et_2P(O)CH_2CH_2P(O)Ph_2$ and denatured protein.[15] Similar results were obtained when the complex was added to solutions of oxidized glutathione, which also contains a disulfide bond, and reduced glutathione was detected as a product. This poses a potential problem with drug delivery, as the Ag(I) complex would need to be protected from endogenous disulfides by a suitable formulation.

*Antimitochondrial Studies*

The yeast *Saccharomyces cerevisiae* is a faculative anaerobe, so cells are able to divide and grow in the absence of oxygen respiration processes, provided glucose is available. In this case, fermentation (or glycolysis) provides sufficient ATP to sustain growth. On the other hand, ATP can be produced in the presence of oxygen by the tricarboxylic acid (TCA, or Krebs) cycle and respiratory chain of the mitochondria. $[Ag(eppe)_2]NO_3$ inhibited the growth of all 15 strains of yeast used in this test. At a concentration of $\leq$ 13 μM (10 μg/mL) on the non-fermentable medium, the cells were unable to process the TCA cycle metabolite, glycerol, indicating that the mitochondria were non-functional, and that no ATP was produced. The dose response depends on the strain, with some strains being inhibited at concentrations as low as 2.5 μM (2 μg/mL). Therefore, $[Ag(eppe)_2]NO_3$ is displaying primary antimitochondrial activity and is affecting mitochondrial function without major disruption of other cellular functions.

The second phase of the testing protocol involved the assessment of the mutagenicity of the silver compound. Here the yeast cultures were grown on a fermentable medium (containing glucose), and were then screened for induction of the well known mitochondrial mutation *petite*. This is a mitochondrial mutation which arises with a high spontaneous frequency (about 1 %) and is characterized by the loss of segments of mitochondrial DNA, presumably through a defect in the replication mechanism.[14] This results in failure of cytochromes $a + a_3$ and $b$ to be synthesized by the mitochondrial system. As noted earlier, growth of *petite* cells can proceed on glucose medium until the glucose is depleted, after which time no growth is possible. This results in small white colonies of the *petite* mutation, rather than the larger creamy colonies of normal cells. Identification of these is unambiguous, the white colour is due to the lack of cytochromes $a + a_3$ and $b$ in these cells. $[Ag(eppe)_2]NO_3$ induced a high frequency of the *petite* mutation (Figure 1). The combination of these two tests indicates that $[Ag(eppe)_2]NO_3$ is an antimitochondrial agent and potential antitumour drug. These studies indicate that the complex is able to selectively target mitochondria, and inhibit the process of respiration. It now remains to be seen if $[Ag(eppe)_2]NO_3$ is able to selectively kill tumour cells as is the case for the metal drug cisplatin, currently in clinical use in cancer treatment.

When aspirin was present in the growth medium with the silver complex, the cells grew normally, and both the toxic and mutagenic effects of the silver compound were lost. Although the reasons for this are unclear, it is an effect that has been observed in previous studies of antimitochondrial compounds in our system.[29] It is suggested that aspirin prevents the penetration of the compounds into the cell by altering membrane permeability, thus protecting the mitochondria.

The related Au(I) complexes, $[Au(eppe)_2]Cl$ and $[Au(dppe)_2]Cl$ showed almost no mitochondrial selectivity in the same screening protocol.[30] Previous studies have shown that

[Au(dppe)$_2$]Cl disrupts the membrane potential in mitochondria and uncouples oxidative phosphorylation, resulting in severe hepato-, cardio- and vascular toxicity.[31 - 33] In isolated rat liver mitochondria, [Au(dppe)$_2$]$^+$ dissipated the membrane potential of the mitochondria, thereby uncoupling oxidative phosphorylation. This is possibly due to increased permeability of the inner membrane to cations and protons.[31] In rabbits this also occurred and ATP synthesis was shown to be reduced and mitochondrial defects were evident, eventually leading to death by cardiotoxicity.[32] Similar results were obtained in a pre-clinical trial of [Au(dppe)$_2$] lactate, which was terminated due to cardio-, hepato- and vascular toxicity.[33] Although mitochondria appear to be a target site for the Au(I) complexes, our studies indicate that these complexes are not mitochondrially selective. It is also noteworthy that the [Au(dppe)$_2$]Cl concentrations of 12.5 - 100 μM used in the isolated rat mitochondria studies were much higher than those used in our yeast studies (≤ 12.5 μM). Therefore it is possible that the acute toxicity observed for [Au(dppe)$_2$]Cl is related to the relatively high dose administered. As [Ag(eppe)$_2$]$NO_3$ is antimitochondrially-active at much lower doses, it may prove to be a more useful chemotherapeutic agent. The higher activity may also be related to the higher water solubility of the silver complex compared to the gold complex, which should result in less accumulation in membranes and therefore greater potency. It is possible that Ag(I) complexes, particularly, [Ag(eppe)$_2$]$NO_3$, which show primary antimitochondrial toxicity, will be useful antitumour agents because they can attack the defective mitochondria of the tumour cells at sufficiently low doses, and destroy the tumour, with less damage being done to healthy cells. It is well known that cancer cells generally have low respiratory rates and high glycolytic activity.[9] The effect of aspirin in preventing the activity of anticancer drugs may be of importance clinically, since this analgesic is liberally prescribed for cancer patients undergoing chemotherapy.

### CONCLUSIONS

The therapeutic use of silver compounds, at present, is limited to topical preparations for antiseptic purposes. The studies reported here show that tetrahedral silver diphosphine complexes, such as [Ag(eppe)$_2$]$NO_3$, show primary antimitochondrial activity, and may be useful as antitumour agents, selectively targeting the respiratory-deficient mitochondria in cancer cells.[9] The choice of ligands and metal appear to be important in controlling this activity. Although the reactivity of the silver compound with components of serum is noted, it may be possible to overcome this with a formulation which delivers the silver complex directly to cancer cells. In addition, the reversal of the effect with aspirin has obvious clinical implications.

### ACKNOWLEDGEMENTS

We thank the Association for International Cancer Research, EC HCM Programme and Royal Society and the Australian NH&MRC (R. Douglas Wright Award to SJBP) for their support of this work, the University of London Inter-collegiate Research Service Biomedical NMR Centre for NMR facilities, the SERC X-Ray Crystallography Service at Cardiff for diffractometry and the Chemical Database Service at Daresbury.

### REFERENCES

1. C.L. Fox, Jr. *Arch. Surg.*, **1968**, 96, 184.
2. P.J. Sadler, *Adv. Inorg. Chem.*, **1991**, 36, 1.
3. T.J. Wlodkowski and H.S. Rosenkrantz, *Lancet II*, **1973**, 739.
   T-W. Chang and L. Weinstein, *J. Infect. Dis.*, **1975**, 132, 79.
   T-W. Chang and L. Weinstein, *Antimicrob. Agents Chemother.*, **1975**, 7, 538.
4. S.J. Berners-Price, R.K. Johnson, A.J. Giovenella, L.F. Faucette, C.K. Mirabelli and P.J. Sadler, *J. Inorg. Biochem.*, **1988**, 33, 285.
5. C.L. Fox, Jr. and S.M. Modak, *Antimicrob. Agents Chemother.*, **1974**, 5, 582.
6. S. Silver and T.K. Misra, *Ann. Rev. Microbiol.*, **1988**, 42, 717.

7. S.J. Berners-Price and P.J. Sadler, *Struct. and Bond. (Berlin)*, **1988**, *70*, 27.
8. C.K. Mirabelli, D.T. Hill, L.F. Faucette, F.L. McCabe, G.R. Gerard, D.B. Bryan, B.M. Sutton, J. O'Leary Bartus, S.T. Crooke and R.K. Johnson, *J. Med. Chem.*,**1987**, 30, 2181.
9. D. Wilkie and K. Fearon, *Mitochondria and Cancer*, in *Achievements in Mitochondrial Research*, (eds. E. Quaglia-Riello et al.), Elsevier, Amsterdam, **1985**, pp. 437 - 444.
10. D. Wilkie, *J. Roy. Soc. Med.*, **1979**, 72, 599.
11. D. Wilkie, *Med. Biol. Illustr.*, **1972**, 22, 119.
12. S.J. Berners-Price, C. Brevard, A. Pagelot and P.J. Sadler, *Inorg. Chem.*, **1986**, 25, 569.
13. A.A. Donopoulos, G. Wilkinson, B. Hussain-Bates and M.B. Hursthouse, *J. Chem. Soc. Dalton Trans.*, **1991**, 1855.
14. G. Bernardi, *Trends. Biochem. Sci.*, **1979**, 4, 197.
15. S.J. Berners-Price, G.R. Gerard, D.T. Hill, B.M. Sutton, P.S. Jarrett, L.F. Faucette, R.K. Johnson, C.K. Mirabelli and P.J. Sadler, *J. Med. Chem.*, **1990**, 33, 1386.
16. G.M. Sheldrick, University of Göttingen, **1986**.
17. G.M. Sheldrick, University of Cambridge, **1993**.
18. N. Walker and D. Stuart, *Acta Cryst.*, **1983**, A39, 158.
19. A. Karaulov, University of Cardiff, **1990**.
20. S.J. Berners-Price, R.E. Norman and P.J. Sadler, *J. Inorg. Biochem.*, **1987**, 31, 197.
21. V. Saboonchian, G. Wilkinson, B. Hussain-Bates and M.B. Hursthouse, *Polyhedron*, **1991**, 10, 737.
22. C.S.W. Harker and E.R.T. Tiekink, *J. Coord. Chem.*, **1990**, 21, 287.
23. Y. Huahui, Z. Lansun, X. Yunjie and Z. Qianer, *J. Inorg. Chem. (Wuji Huaxue Xuebao)*, **1992**, 8, 65.
24. G.A. Bowmaker, P.C. Healy, L.M. Engelhardt, J.D. Kildea, B.W. Skelton and A.H. White, *Aust. J. Chem.*, **1990**, 43, 1697.
   F.A. Cotton and R.L. Luck, *Acta Cryst.*, **1989**, C45, 1222.
   L.M. Engelhardt, C. Pakawatchai, A.H. White and P.C. Healy, *J. Chem. Soc. Dalton Trans.*, **1985**, 125.
   C. Pelizzi, G. Pelizzi and P. Tarasconi, *J. Organomet. Chem.*, **1984**, 277, 29.
25. D. Franzoni, G. Pelizzi, G. Predieri, P Tarasconi and C. Pelizzi, *Inorg. Chim. Acta*, **1988**, 150, 279.
26. S.J. Berners-Price, C. Brevard, A. Pagelot and P.J. Sadler, *Inorg. Chem.*, **1985**, 24, 4278.
27. E.L. Muetterties and C.W. Alegranti, J. *Am. Chem. Soc.*, **1972**, 94, 6386.
28. N.A. Malik, G. Otiko and P.J. Sadler, *J. Inorg. Biochem.*, **1980**, 12, 317.
   C.F. Shaw, III, A.A. Isab, J.D. Hoeschele, M. Starich, J. Locke, P. Schulteis and J. Xiao, *J. Am. Chem. Soc.*, **1994**, 116, 2254.
29. D.C. Collier and D. Wilkie, unpublished results.
30. S.J. Berners-Price, D.C. Collier, P.J. Sadler, R.E. Sue and D. Wilkie, unpublished results.
31. G.D. Hoke, G.F. Rush, G.E. Bossard, J.V. McArdle, B.D. Jensen and C.K. Mirabelli, *J. Biol. Chem.*,**1988**, 263, 11203.
32. G.D. Hoke, R.A. Macia, P. Meunier, P.J. Bugelski, C.K. Mirabelli, G.F. Rush and W.D. Matthews, *Toxicol. and Appl. Pharm.*, **1989**, 100, 293.
33. G.F. Rush, D.W. Alberts, P. Meunier, K. Leffler, and P.F. Smith, *Toxicologist*, **1987**, 7, 59.

**Received: December 1, 1994 - Accepted: January 6, 1994 - Received in revised camera-ready format: January 30, 1995**

